# BMP4 upregulates glycogen synthesis through the SMAD/SLC2A1 (GLUT1) signaling axis in hepatocellular carcinoma (HCC) cells

**DOI:** 10.1186/s40170-023-00310-6

**Published:** 2023-07-13

**Authors:** Jiamin Zhong, Luyao Tian, Yannian Gou, Piao Zhao, Xiangyu Dong, Meichun Guo, Guozhi Zhao, Aohua Li, Ailing Hao, Tong-Chuan He, Jiaming Fan

**Affiliations:** 1 Ministry of Education Key Laboratory of Diagnostic Medicine, Chongqing, China; 2 Department of Clinical Biochemistry, College of Laboratory Medicine, Chongqing Medical University, No. 1 Medical School Road, Yuzhong District, Chongqing, 400016 China; 3 Molecular Oncology Laboratory, Department of Orthopaedic Surgery and Rehabilitation Medicine, The University of Chicago Medical Center, 5841 South Maryland Avenue, MC 3079, Chicago, IL 60637 USA; 4 Department of Orthopedics, The First Affiliated Hospital of Chongqing Medical University, Chongqing, 400016 China; 5 Department of Urology, The First Affiliated Hospital of Chongqing Medical University, Chongqing, 400016 China

**Keywords:** HCC, BMP4, SLC2A1, Smad signal pathway, Glycogen synthesis

## Abstract

**Background:**

Excessive hepatic glycogen accumulation benefits tumorigenesis and cancer cell survival. We previously reported that BMP4 has the strongest ability to promote glycogenesis among the 14 BMPs in hepatocytes and augmented hepatocellular carcinoma (HCC) cell survival under hypoxia and hypoglycemia conditions by promoting the glycolysis pathway. However, the mechanism underlying BMP4’s effect on glycogenesis in HCC remains elusive.

**Methods:**

The expression of BMP4 and SLC2A1 were acquired by analyzing the TCGA-LIHC dataset, as well as by immunohistochemical analysis of the 40 pairs of human HCC samples and para-tumor tissues. Gene expressions were detected by qPCR, immunoflurorescence staining, and Western blotting. Overexpression and silencing of BMP4 were accomplished through adenoviruses Ad-B4 and Ad-siB4 infection. Hepatic glycogen was detected by PAS staining. SLC2A1 (GLUT1) function was blocked by the inhibitor BAY-876. ChIP assay was used to determine the binding of SMADs to the promoter region of SLC2A1 in HCC cells. Lastly, the in vivo effect of BMP4-regulated SLC2A1 on HCC tumor growth was assessed in a xenograft model of HCC.

**Results:**

The elevated expression of BMP4 in HCC tumor tissues was highly correlated with hepatic glycogen accumulation in clinical samples. SLC2A1 was highly expressed in HCC tumor tissue and correlated with clinical stage and prognosis. Exogenous BMP4 augmented glycogen accumulation and upregulated the expression of glycogen synthesis-related genes in Huh7 and HepG2 cells, both of which were effectively blunted by SLC2A1inhibitor BAY-876. In mechanism, BMP4 activated SMAD5 to regulate the promoter of SLC2A1to enhance its expression. The in vivo xenograft experiments revealed that BMP4 promoted glycogen accumulation and tumor growth, which were effectively diminished by BAY-876.

**Conclusion:**

These results demonstrate that BMP4 upregulates glycogen synthesis through the SMAD/SLC2A1 (GLUT1) signaling axis in HCC cells, which may be exploited as novel therapeutic targets for HCC treatment.

**Supplementary Information:**

The online version contains supplementary material available at 10.1186/s40170-023-00310-6.

## Introduction

It is well-known that cancer cells can undergo metabolic reprograming to adapt hypoxia microenvironment for survival and growth. It has been demonstrated that even under normoxia condition, tumor cells depend on glycolysis rather than oxidative phosphorylation to convert glucose into lactate, an inefficient pathway of ATP production, to survive and progress [1–4]. Since glucose is at the center of carbohydrate metabolism, and glycogen is readily obtainable energy reservoir of various tissues and cells during metabolic processes, it is important to investigate whether the regulation of glycogen synthesis is exploited by cancer cells to meet energy requirements [5].

Hepatocellular carcinoma (HCC) is the most predominant type of primary liver cancer and the third leading cause of cancer-related mortality in the world [6, 7]. The liver is the most glycogen-enriched organ in the body and maintains glycogen metabolic homeostasis by regulating key enzyme activity for glycogenesis and glycogenolysis. Glycogen accumulation is a key carcinogenic event in the malignant transformation of the liver [8]. It has been recently reported that excessive glycogen accumulation was found in early-stage hepatic tumor lesions and small tumors, which drove tumorigenesis and enhanced cancer cell survival [9, 10]. Meanwhile, earlier studies demonstrated that a reduction in the activities of glucose-6-phosphatase (G6Pase) and glycogen phosphorylase (GP), a key enzyme of glycogenolysis, contributed to a statistically increase in hepatic glycogen accumulation in rats with cirrhosis [5]. Interestingly, it was reported that despite the decrease in hepatic glycogen content in CCl4-induced cirrhotic rats, the total hepatocyte glycogen synthase activity was enhanced and the reason may be that the loss of hepatocytes observed in CCl4-induced cirrhotic rats leads to a reduce in hepatic glycogen content per gram of liver in cirrhotic rats [11]. Nonetheless, it remains to be fully understood about how glycogen synthesis and accumulation is dysregulated during tumorigenesis and cancer progression, which may be targeted as novel cancer drug targets.

Bone morphogenetic protein 4 (BMP4) belongs to the TGF-β family and initiates downstream signaling by binding to TGFβ type I or type II serine/threonine kinase receptors and forming a heterotetrameric complex [12]. BMP4 is an important regulator of adipogenesis associated with obesity and diabetes [13–17]. Hence, understanding the possible role of BMP4 in regulating hepatic glycometabolism during the development and progression of hepatocellular carcinoma may aid us to identify new therapeutic targets for HCC treatment. We previously demonstrated that among the 14 types of BMPs (i.e., from BMP2 to BMP15), BMP4 had the most potent capability to promote glycogen accumulation in primary mouse hepatocytes, followed by BMP2, BMP7, and BMP8 [18, 19]. However, the mechanism underlying BMP4’s effect on glucose metabolism remains elusive.

In this study, we found that BMP4 expression was correlated with glycogen accumulation and SLC2A1(GLUT1) expression in clinical HCC samples. Exogenous BMP4 augmented glycogen accumulation and upregulated the expression of glycogenesis-related and glycogenolysis-related genes in HCC cells, both of which were effectively blunted by SLC2A1inhibitor BAY-876. Furthermore, our ChIP assay revealed that BMP4-activated SMAD5 was able to bind to the promoter of SLC2A1 to enhance its expression. Using a xenograft model of HCC, we found that BMP4 promoted glycogen accumulation and tumor growth, which could be effectively blocked by BAY-876. Thus, our results demonstrate that BMP4 upregulates glycogen synthesis through the SMAD/SLC2A1 (GLUT1) signaling axis in HCC cells.

## Materials and methods

### The use of clinical samples

A panel of 40-paired HCC tumor and adjacent non-tumor samples was obtained from the Department of Pathology, The First Affiliated Hospital of Chongqing Medical University. The use of the human clinical samples in this study was approved by the Research Ethics and Regulations Committee of Chongqing Medical University, Chongqing, China.

### Cell culture and chemicals

HEK-293 derived 293pTP and RAPA cells were previously described [20, 21]. Human hepatocyte line LO2, Human HCC lines Huh7, MHCC97H, HepG2, and Hep3B were obtained from the Key Laboratory of Clinical Laboratory Diagnostics of Ministry of Education, Chongqing Medical University. All cells were cultured in DMEM supplemented with 10% fetal bovine serum (Lonsera, Cat: S711-0015, Uruguay) containing 100 units of penicillin and 100 µg of streptomycin in a 5% CO_2_ incubator at 37 °C. BAY-876 was purchased from Selleckchem (Cat: S8452, Houston, TX, USA). LDN193189 (Cat: HY-12071, MCE, USA). Unless otherwise stated, all chemicals were purchased from Sigma-Aldrich (St Louis, MO, USA), Thermo Fisher Scientific (Waltham, MA, USA), or Solarbio (Beijing, China).

### Construction and amplification of recombinant adenoviral vectors Ad-B4, Ad-siB4, Ad-GFP, and Ad-RFP

The AdEasy technology was used to generate recombinant adenovirus as described [22–24]. The construction and amplification of Ad-B4 that overexpresses human BMP4 was described previously [25]. The construction and amplification of Ad-siB4 that silences the human *BMP4* was carried by using our recently developed methods as described [26–28]. Both Ad-GFP and Ad-RFP were used as controls [29]. Ad-B4 also expresses GFP, while Ad-siB4 co-expresses RFP. Fluorescence signals were documented under a fluorescence microscope at 36 h after infection. For all adenoviral infections, polybrene (8 µg/ml) was added to enhance infection efficiency as reported [19, 30].

### H & E and immunohistochemical (IHC) staining

The tumor tissue sections were deparaffinized, rehydrated, and subjected to H & E staining and IHC staining as described [19, 29, 30]. Briefly, the tissue sections were deparaffinized, rehydrated, antigen-retrieval treated, blocked, and incubated overnight with appropriate dilutions of primary antibodies against BMP4 (1:50 dilution; Bimake; Cat# A5543), SLC2A1 (1:100 dilution; Proteintech; Cat# 21829-1-AP), SMAD5 (1:50 dilution; Bimake; Cat# A5511), or p-SMAD5 (1:200 dilution; Abcam; Cat# ab92698), followed by stained with biotin-labeled goat anti-rabbit IgG and horseradish peroxidase-conjugated-labeled streptavidin. IHC scores and PAS staining were calculated based on the percentage of positive cells and the degree of staining. The IHC staining scores were defined into 5 levels as follow: Level 1, positive cells accounting for ≤ 20%; Level 2, positive cells accounting for 20–40%; Level 3, positive cells accounting for 40–60%; Level 4, positive cells accounting for 60–80%; and Level 5, positive cells accounting for > 80%. Samples with IHC staining scores < Level 3 were designated as low expression groups, while samples with ≥ Level 3 were designated as high expression groups. The scoring outcomes of HCC tissue and adjacent liver non-tumor tissue samples for anti-BMP4 and anti-SLC2A1 IHC are shown in Table S1. Staining results were recorded under a bright #field microscope (magnification, ×100 or ×400). Each assay condition was done in triplicate. Representative results are shown.

### Total RNA extraction and touchdown-quantitative real-time PCR (TqPCR)

The total RNA was isolated from the cultured cells using TRIZOL Reagent (Invitrogen, China) as described [31, 32]. Briefly, Huh7 and HepG2 cells treated with various conditions were lysed in TRIZOL Reagent for total RNA isolation. The total RNA was subjected to reverse transcription with hexamer and M-MuLV reverse transcriptase (New England Biolabs, Ipswich, MA). The cDNA products were used as PCR templates. Gene-specific PCR primers were designed by using Primer 3 program. All primer sequences are shown in Table S2. TqPCR was carried out by using 2× SYBR Green qPCR Master Mix (Bimake, Shanghai, China) on the CFX-Connect unit (Bio-Rad Laboratories, Hercules, CA) as described [32]. All TqPCR reactions were done in triplicate. *GAPDH* was used as a reference gene. Quantification of gene expression was carried out by using the 2^−ΔΔCq^ method as described [31].

### Western blotting analysis

Western blotting was performed as previously described [33]. Huh7 and HepG2 cells were treated with various conditions and lysed in RIRA Lysis Buffer containing phosphatase inhibitors and protease inhibitors. Cell lysates were separated by 10% SDS-PAGE and transferred to PVDF membranes. The proteins of interest were assessed by being incubated with the following primary antibodies at 4 °C overnight: anti-GYS1(1:500; Abcam; Cat#ab40810), anti-BMP4 (1:1000; Bimake; Cat# A5543), anti-SLC2A1 (1:1000; Proteintech; Cat# 21829-1-AP), anti-SMAD5 (1:1000, Bimake; Cat# A5511), anti-p-SMAD5 (1:1000; Abcam; Cat# ab92698), and anti-β-Actin (1:2000, Proteintech; Cat# 60008-1-Ig). Subsequently, the membranes were incubated with HRP-conjugated goat anti-mouse or anti-rabbit secondary antibody for 1 h at 37 °C, respectively (1:5000; ZSGB-BIG, Cat# ZB-2306 or 2305). Lastly, the proteins were detected on the Bio-Rad ChemiDoc Imager (Hercules, CA) by using an Enhanced Chemiluminescence (ECL) kit (Millipore, USA). Each assay condition was done in triplicate. Representative results are shown. The original images of WB were shown in Supplementary Fig. S1.

### Immunofluorescence (IF)

The IF staining was carried out as reported [29]. Huh7 and HepG2 cells were seeded in 24-well plates onto coverslips and treated with various conditions, followed by being fixed with 4% paraformaldehyde at RT for 20 min and blocked with 5% bovine serum albumin (BSA) for 30 min at RT. The coverslips were incubated with the primary antibody anti-SLC2A1 (1:100; Proteintech; Cat# 21829-1-AP) at 4 °C overnight and incubated with Rhodamine (TRITC)–conjugated goat anti-rabbit IgG (H + L) (1:200; Proteintech; Cat# SA00007-2) and CoraLite488-conjugated goat anti-rabbit IgG(H + L) (1:200; Proteintech; Cat# SA00013-2) for 2 h at RT. The nuclei were counterstained with DAPI (10 µg/mL) for 5 min at RT. Minus primary antibody only was used as a negative control. Fluorescent images were obtained with a laser confocal microscope (Leica TCS SP8, magnification × 400). Each assay condition was done in triplicate. Representative results are shown.

### Glycogen accumulation and quantitative analyses

PAS staining was conducted using Glycogen Periodic Acid Schiff (PAS/hematoxylin) Stain Kit as described [34]. The staining results were recorded under a bright field microscope (magnification, ×100 or ×400). Glycogen quantification assay was performed by using the glycogen assay kit (Solarbio, Glucogen Assay Kit; Cat# BC0340). Each assay condition was done in triplicate. Representative results are shown.

### WST-1 cell proliferation assay

WST-1 assay was conducted as described [35]. Briefly, the Huh7 and HepG2 cells were plated in 96-well plates at a density of 2000 cells per well and treated with different conditions. At the indicated time points, the Premixed WST-1 Reagent (Clontech, Mountain View, CA) was added, followed by incubating at 37 °C for 120 min and reading absorbance at 450 nm. Each assay condition was done in triplicate.

### Chromatin immunoprecipitation (ChIP)

Consensus Smad1/Smad5 binding sites were previously characterized [36, 37]. Potential binding motifs for *Smad1/5* at the *SLC2A1* promoter were predicted by using the JASPAR program (https://jaspar.genereg.net/. Accessed 20 May 2022) [38, 39]. ChIP assay was carried out by using the ChIP Assay Kit (Beyotime, China, # P2078) as previously described [40]. Briefly, Huh7 and HepG2 cells were infected with Ad-B4 for 36 h, and formaldehyde cross-linked, which was terminated by adding glycine. The cross-linked cells were collected by centrifugation at 4 °C and then sonicated in an ice bath. Ultrasonic samples were centrifuged at 12,000 g and then incubated with protein A + G agarose/salmon sperm DNA with occasional agitations at 4ºC for 30 min, followed by centrifugation to remove non-specific binding DNA/protein complexes. The cleared supernatant samples were incubated with Anti-SMAD5 (1:20 dilution; Bimake; Cat# A5511) or goat IgG to pull down the protein-DNA complex at 4 °C overnight. The complexes were captured by protein A + G agarose/salmon sperm DNA, followed by being washed sequentially in different wash buffers. The pulled down precipitates (i.e., DNA–protein complexes) were dissolved in freshly prepared elution buffer (1% SDS, 0.1 M NaHCO3). The mixtures were heated at 65 °C for 4 h to reverse the formaldehyde cross-linking in a high salt buffer (0.2 M NaCl). The pulled down genomic DNA was isolated by using the DNA Purification Kit (Beyotime# D0033) for PCR. The presence of SLC2A1 promoter fragments was detected by semi-quantitative PCR using and the primer sequences are listed in Table S3.

### Xenograft tumor model of human HCC in vivo

The use and care of experimental animals were approved by the Research Ethics and Regulations Committee of Chongqing Medical University, Chongqing, China. All experimental procedures followed the approved guidelines. Athymic nude mice were obtained from and housed in the Experimental Animal Research Center of Chongqing Medical University. The cancer cell implantation experiments were carried out as previously described [40]. Briefly, Huh7 cells were infected with Ad-B4 and Ad-GFP, and collected and resuspended in sterile PBS for subcutaneous injection into the flanks of athymic nude mice (5–6 weeks old, male, 5 × 10^6^ cells/injection, 4 injections per mouse, and 3 mice per group). The mice in the Ad-B4 + BAY-876 group also were administered through gavage with BAY-876 (2.5 mg/kg) in 0.25% carboxymethyl cellulose (CMC) every 3 days, while the mice in the Ad-B4 group and Ad-GFP group were administered through gavage with equal volumes 0.25% CMC. Tumor volumes were measured every 7 days. At 28 days after injection, mice were sacrificed, and the subcutaneous tumor masses were retrieved. The tumor volume of tumor was calculated according to the formula LW2/2, where L is the length and W is the width of the tumor. The subcutaneous tumor masses were subjected to paraffin embedding and histologic and IHC staining.

### Statistical analysis

All experiments performed at least three times and/or repeated in three independent batches. Data were analyzed using GraphPad Prism 8 and presented as mean ± standard deviation (SD). Statistical significance was confirmed by one-way analysis of variance and the student’s *t* test for the comparisons between groups. A value of *P* < 0.05 was considered statistically significant.

## Results

### High expression of BMP4 is significantly correlated with the expression of glucose transporter SLC2A1 in HCC

**Fig. 1:**
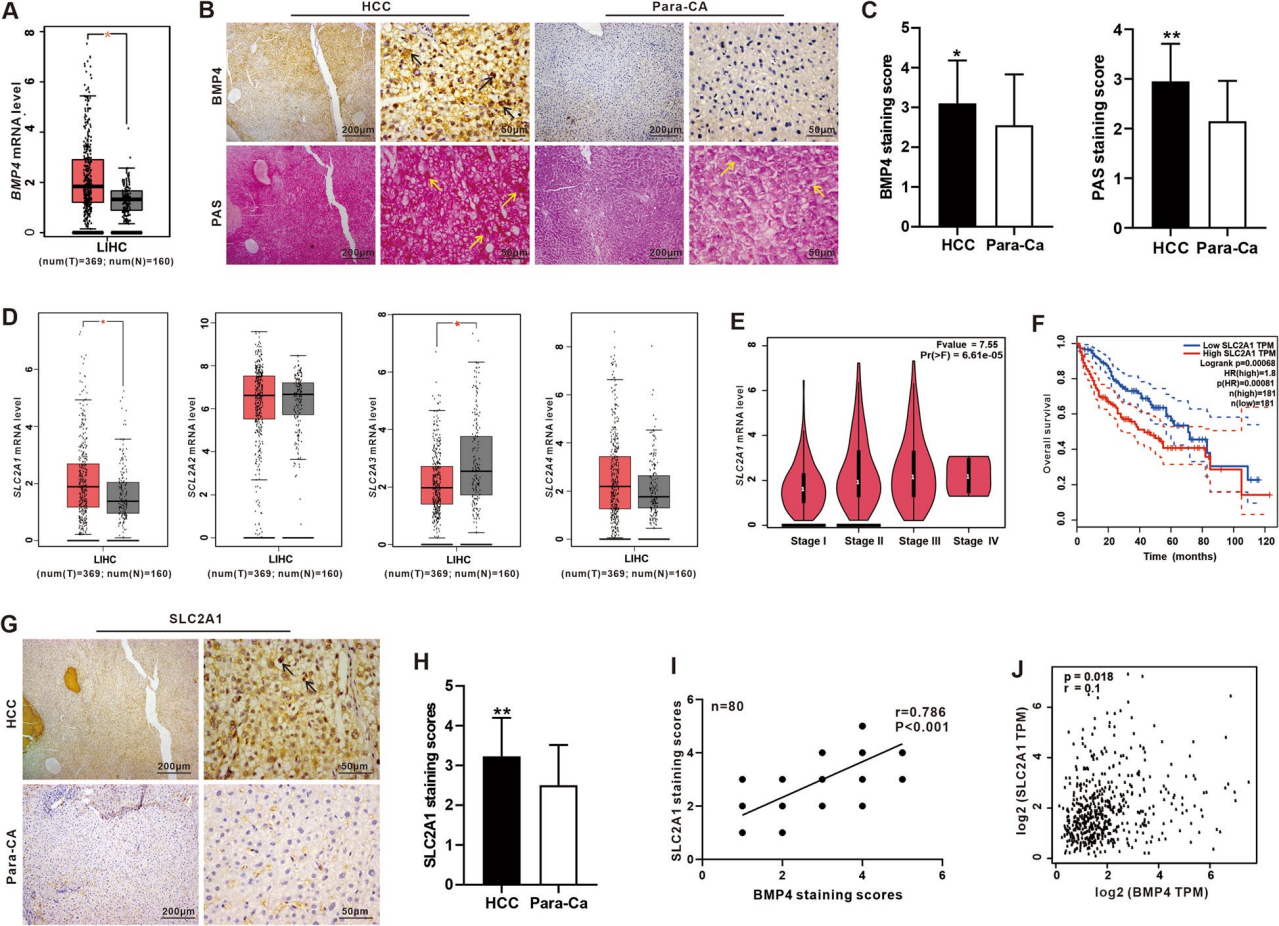
The expression of BMP4 and SLC2A1 is elevated and positively correlated in HCC. **A** Comparative analysis of the expression of *BMP4* between tumor tissues (T, *n* = 369) and adjacent non-tumorous liver tissues (N, *n* = 160) from TCGA. *P < 0.05, T group vs. N group. **B** IHC staining analysis of BMP4 protein expression and PAS staining in HCC tissues and adjacent non-tumorous liver tissue (Para-CA) of 40-paried patient samples. Positive staining was indicated by arrows. **C** The scores of IHC of BMP4 and PAS in B were judged by degree and range of positive staining. *P < 0.05, HCC group vs. para-Ca group. **D** Comparative analysis of the expression of *SLC2A1, SLC2A2, SLC2A3*, and *SLC2A4* between tumor tissues (T, *n* = 369) and adjacent non-tumorous liver tissues (N, *n* = 160) from TCGA. *P < 0.05, T group vs. N group. **E** Violin plot analysis of the *SLC2A1* expression level from stage I to stage IV in LIHC from TCGA. **F** The overall survival time of HCC patients related to SLC2A1 was analyzed by Kaplan-Meier survival analysis from TCGA. **G** IHC staining of SLC2A1 expression in HCC tissues and adjacent non-tumorous liver tissue (para-CA) of 40 paired HCC specimens. Positive staining was indicated by arrows. **H** The scores of IHC were judged by degree and range of positive staining. *P < 0.05, HCC group vs. para-Ca group. **I** Pearson’s correlation analysis of IHC scores results between BMP4 and SLC2A1 expression of 40 paired HCC specimens (**P < 0.01). **J** Pearson’s correlation analysis between *Bmp4* and *SLC2A1* mRNA expression from GTE

In our previous study, we found that BMP4 augments the survival of hepatocellular carcinoma (HCC) cells under hypoxia and hypoglycemia conditions by promoting the glycolysis pathway [30]. However, the effect of BMP4 on glycogen synthesis in hepatoma cells is still unknown. Through bioinformatics analysis of the dataset from the TCGA Database (via the website: http://gepia.cancer-pku.cn/detail.php. Accessed 20 Aug 2021) [41], we first demonstrated that the expression of *BMP4* was higher in the HCC tissues than that in the adjacent non-tumor tissues (Fig. 1A). Using the clinical HCC samples, we confirmed that both BMP4 expression and glycogen content significantly increased in HCC tissues, compared with that in adjacent non-tumor tissues by IHC and PAS staining assays (Fig. 1B, C).

Glucose transporters include SLC2A1, SLC2A2, SLC2A3, and SLC2A4, and we found the expression of *SLC2A1* was significantly upregulated in HCC tumor, contrast with that in adjacent non-tumor tissues (from TCGA Database) (Fig. 1D). We also found a progressive increase in the expression of *SLC2A1* from HCC stage I to IV (from TCGA Database) (Fig. 1E), and the median overall survival time of SLC2A1 high expression group was significantly shorter than that of SLC2A1 low expression group (from TCGA Database) (Fig. 1F). IHC staining of clinical HCC samples showed that SLC2A1 was high expression in tumor tissues in contrast with that in adjacent non-tumor tissues (Fig. 1G, H). Pearson’s correlation analysis of IHC results confirmed a positive correlation between BMP4 and SLC2A1 expression by IHC staining scores (Fig. 1I). Pearson’s correlation analysis showed a positive association with BMP4 and SLC2A1 aberrant expression (from TCGA Database) (Fig. 1J). Thus, these results indicate SLC2A1 is highly expressed in HCC and closely related to tumor clinical stage and survival rates, which is also positively associated with highly expressed BMP4.

### BMP4 promotes glycogen synthesis in Huh7 and HepG2 cells

**Fig. 2:**
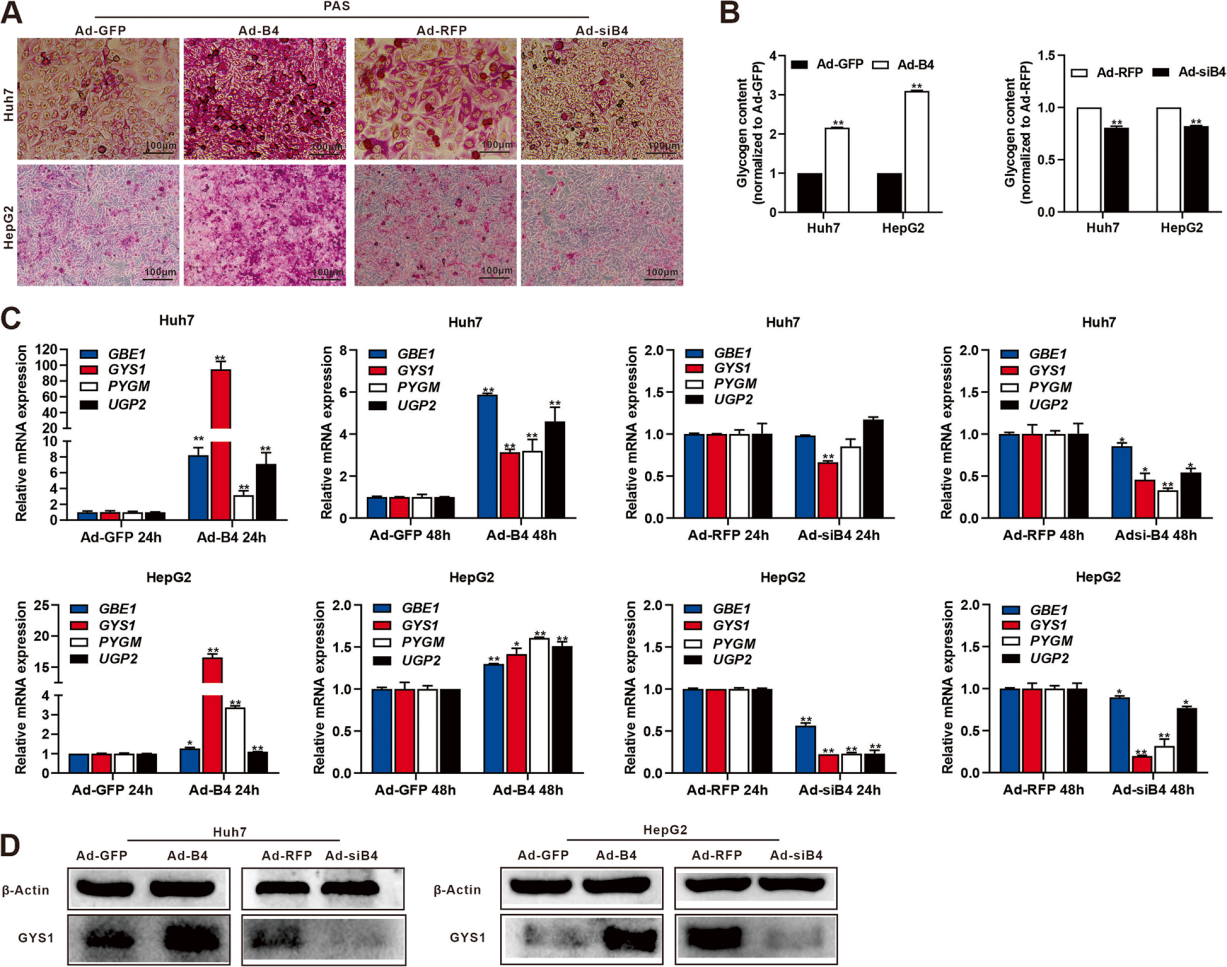
BMP4 promotes glycogen synthesis in Huh7 and HepG2 cells.** A** Huh7 and HepG2 cells were treated with Ad-B4, Ad-GFP, Ad-siB4, or Ad-RFP to overexpression or silence BMP4, respectively, and PAS staining was subjected to evaluate glycogen accumulation after 72 h. **B** Glycogen assay was subjected to evaluate glycogen content of 2A. **P < 0.01, *P < 0.05, Ad-B4 group vs Ad-GFP group, Ad-siB4 group vs Ad-RFP group. **C** TqPCR analysis was carried out to detect the expression of key enzymes of glycogenesis *GBE1*, *GYS1*, *UGP2*, and glycogenolysis *PYGM* at 24 h and 48 h of 2A. **P < 0.01, **P < 0.05, Ad-B4 group vs Ad-GFP group, Ad-siB4 group vs Ad-RFP group. **D** Western blot was used to analyze the expression of GYS1 in Huh7 and HepG2 cells after 72 h

**Fig. 3:**
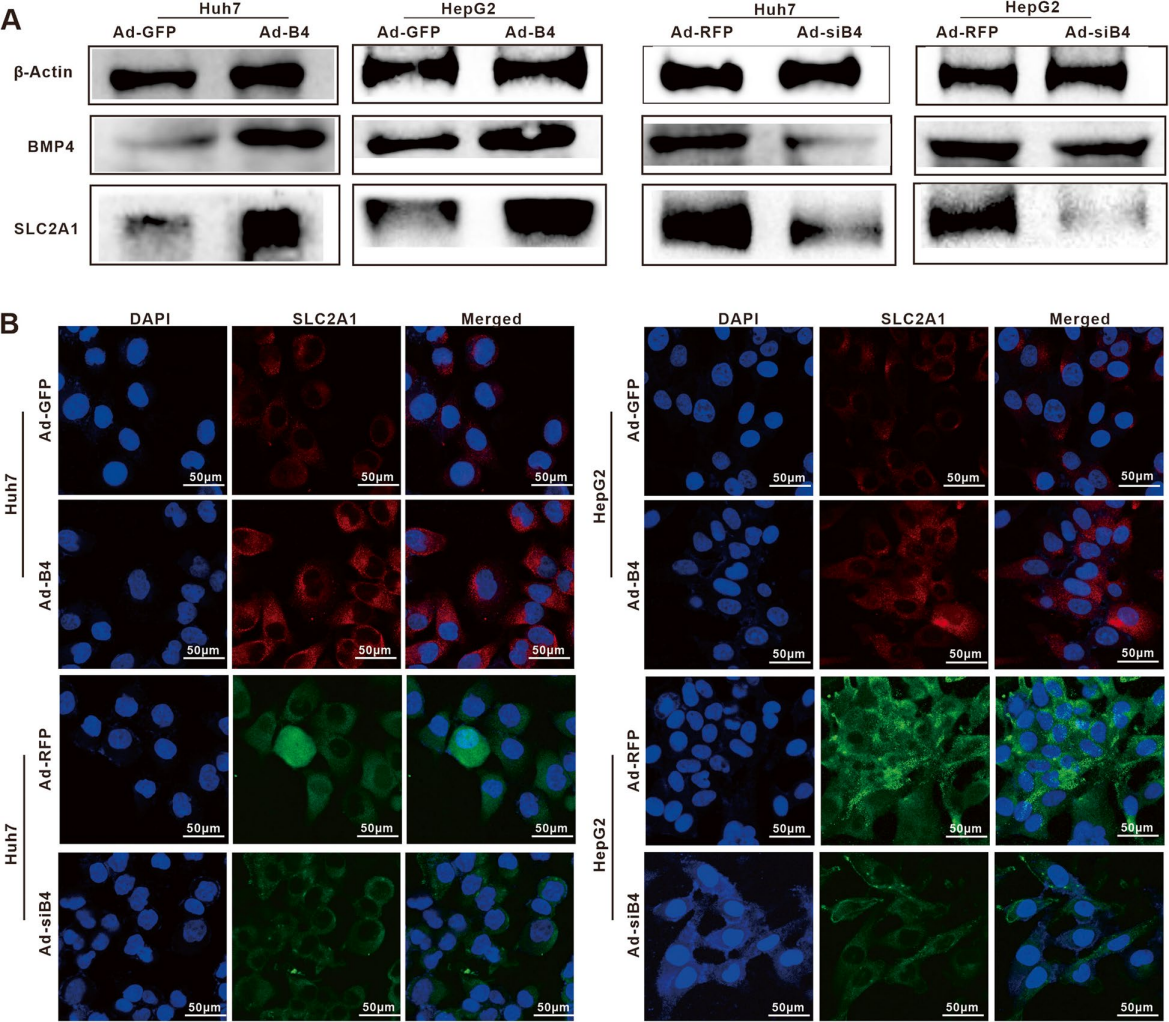
BMP4 promotes SLC2A1 expression in Huh7 and HepG2 cells. **A** Huh7 and HepG2 cells were treated with Ad-B4, Ad-GFP, Ad-siB4, and Ad-RFP to overexpression or silence BMP4, respectively, western blot was used to analyze the expression of BMP4 and SLC2A1 in HepG2 and Huh7 cells after 72 h. **B** IF staining was used to show the expression of SLC2A1 in Huh7 and HepG2 cells after 48 h in **A**. The nuclei were counter-stained with DAPI

We further found that BMP4 could be detected in Lo2, Huh7, MHCC97H, HepG2, and Hep3B cells, whereas SLC2A1 was high expression in Huh7 and HepG2 cells (Fig. S2A). Huh7 and HepG2 were chosen for further study. Using recombinant adenoviruses Ad-B4 and Ad-siB4, which overexpresses or silences BMP4, respectively, we infected Huh7 and HepG2 cells with high efficiency as the fluorescence signals were readily detected at 36 h after infection (Fig. S2B). The expression of BMP4 was detected at 72 h after infection (Fig. 3A). We found that BMP4 increased the glycogen accumulation as confirmed by PAS staining (Fig. 2A), and the quantitative glycogen content assays (Fig. 2B) in Huh7 and HepG2 cells. Furthermore, TqPCR analysis showed that glycogenesis-related genes *GBE1*, *GYS1*, *UGP2*, and glycogenolysis-related genes *PYGM* were significantly upregulated by BMP4 both at 24 h and 48 h compared with Ad-GFP control group, while conversely, silencing *BMP4* effectively inhibited the expression of the above genes both at 24 h and 48 h compared with Ad-RFP control group in the Huh7 and HepG2 cells (Fig. 2C). Western blotting showed that BMP4 upregulated the expression of GYS1 while effectively inhibited the expression of GYS1 after silencing *BMP4* in the Huh7 and HepG2 cells after 72 h (Fig. 2D)*.* Taken together, these results indicate that BMP4 promotes the glycogen accumulation and content by increasing glycogen synthesis in Huh7 and HepG2 cells.

### BMP4 upregulates the expression of SLC2A1 in Huh7 and HepG2 cells

**Fig. 4:**
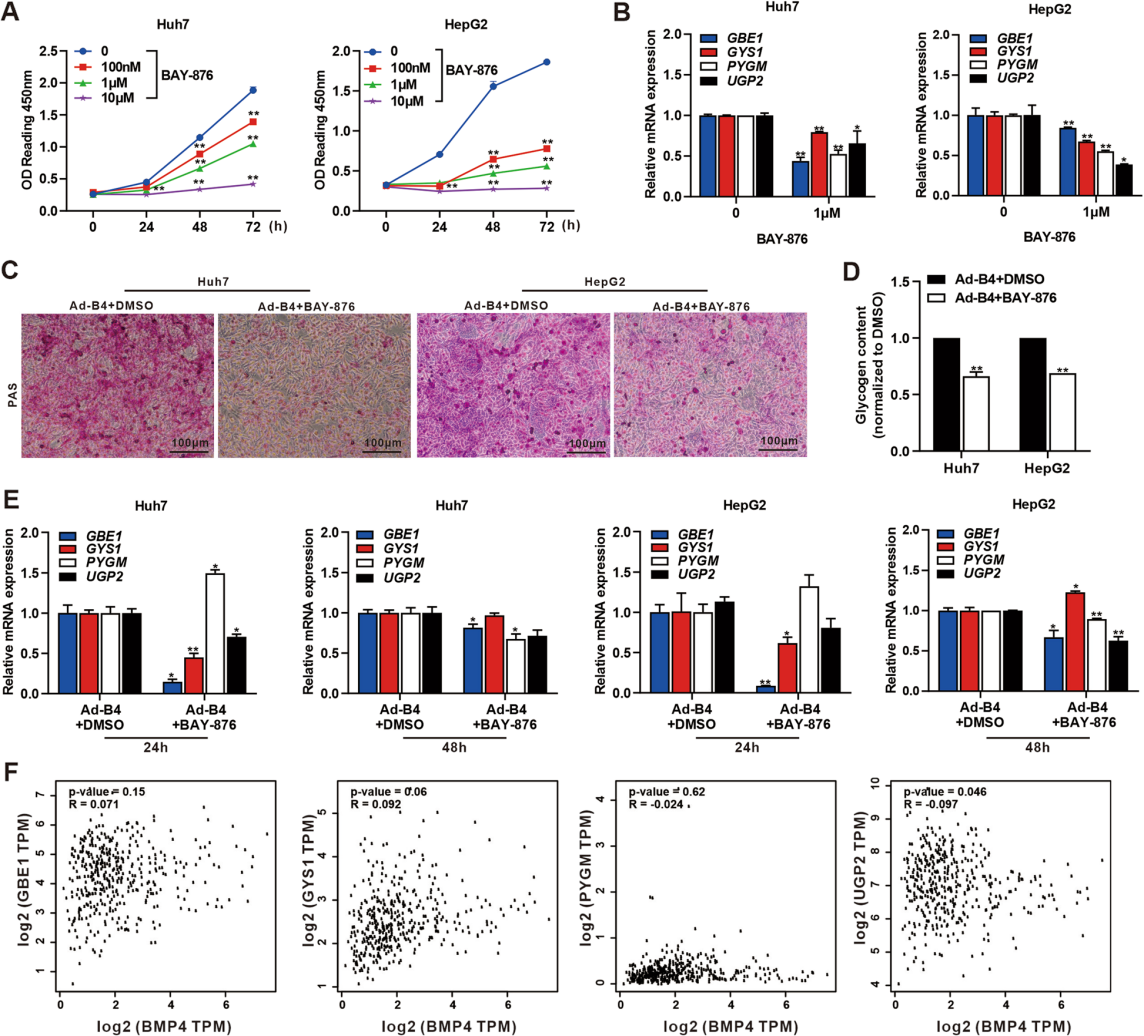
BMP4-induced glycogen synthesis can be blocked by SLC2A1 inhibitor in Huh7 and HepG2 cells. **A** Different doses of BAY-876 were used to treat Huh7 and HepG2 cells, and cell proliferation was assessed at 0, 24, 48, and 72 h by WST-1. **B** 1 μm BAY-876 or equal volume DMSO was used to treated Huh7 and HepG2 cells, and TqPCR analysis was carried out to detect the expression of key enzymes of glycogenesis *GBE1*, *GYS1*, *UGP2*, and glycogenolysis *PYGM* at 48 h. **P < 0.01, **P < 0.05, 0 (DMSO) group vs 1 μm group. **C** Ad-B4 was used to infect Huh7 and HepG2 cells and treated with 1 μm BAY-876 or equal volume DMSO, and PAS staining was subjected to evaluate glycogen accumulation after 72 h. **D** Ad-B4 was used to infect Huh7 and HepG2 cells and treated with 1 μm BAY-876 or equal volume DMSO. The glycogen content assay was subjected to evaluate glycogen level after 72 h. **P < 0.01, Ad-B4 + DMSO group vs Ad-B4 + BAY-876 group. **E** Huh7 and HepG2 cells were infected with Ad-B4 and treated with 1 μm Bay-876 or equal volume DMSO, respectively. TqPCR analysis was carried out to detect the expression of *GBE1*, *GYS1*, *PYGM*, and *UGP2* at 24 h and 48 h, respectively. **P < 0.01, *P < 0.05, Ad-B4 + DMSO group vs Ad-B4 + BAY-876 group. **F** The correlation of BMP4 with *GBE1*, *GYS1*, *PYGM*, and *UGP2* in LIHC data from TCGA tumor

We next found BMP4-upregulated expression of *SLC2A1, SLC2A2, SLC2A3,* and *SLC2A4* by qPCR (Fig. S2C), and BMP4-upregulated expression of SLC2A1 was also confirmed by Western blotting analysis in Huh7 and HepG2 cells (Fig. 3A). Furthermore, IF staining revealed the BMP4-upregulated expression of SLC2A1 in cell membrane and cytoplasm of Huh7 and HepG2 cells (Fig. 3B). BAY-876 is an orally active and selective SLC2A1 inhibitor. We found that BAY-876 inhibited the proliferation of Huh7 and HepG2 cells in a dose-dependent fashion in a range from 0 to 10 μM (Fig. 4A). TqPCR analysis showed BAY-876 (at 1 μM) significantly inhibited the expression of glycogenesis-related genes *GBE1*, *GYS1*, *UGP2*, and glycogenolysis-related genes *PYGM* (Fig. 4B). BAY-876 significantly reduced the glycogen accumulation/content induced by BMP4 (Fig. 4C, D), and the BMP4-upregulated expression of *GBE1*, *GYS1*, *UGP2*, and *PYGM* was downregulated by BAY-876 in varying degrees in Huh7 and HepG2 cells (Fig. 4E). We analyzed the data from TCGA (via the gepia website: http://gepia.cancer-pku.cn/detail.php) and found a positive association with *BMP4* and *UGP2* in LIHC (Fig. 4F). Collectively, these results demonstrate that the expression of SLC2A1 in Huh7 and HepG2 cells is positively regulated by BMP4.

### BMP4 facilitates the transcription of *SLC2A1* via the canonical SMAD signaling pathway

**Fig. 5:**
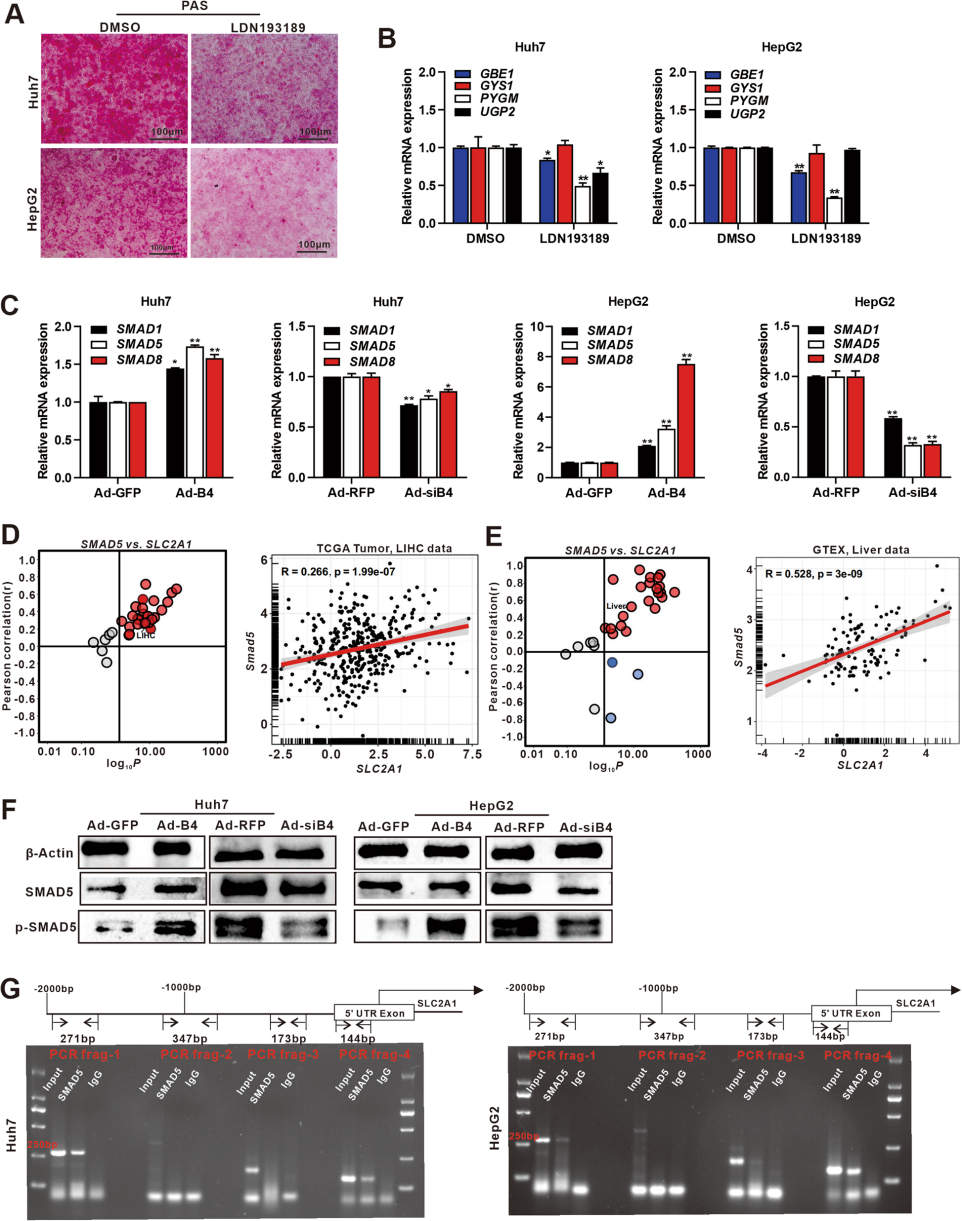
BMP4 regulates SLC2A1 expression through canonical SMAD signaling in Huh7 and HepG2 cells. **A** Huh7 and HepG2 cells were treated with 1 μM LDN193189 or equal volume DMSO, and PAS staining was subjected to evaluate glycogen accumulation after 72 h. **B** Huh7 and HepG2 cells were treated with 1 μM LDN193189 or equal volume DMSO, respectively. TqPCR analysis was carried out to detect the expression of *GBE1*, *GYS1*, *PYGM*, and *UGP2* at 48 h. **P < 0.01, *P < 0.05, LDN193189 group vs DMSO group. **C** Huh7 and HepG2 cells were treated with Ad-B4, Ad-GFP, Ad-siB4, and Ad-RFP to overexpress or silence BMP4, respectively. TqPCR analysis was used to evaluate the expression of *SMAD1/5/8* at 48 h. **D** The correlation of *SMAD5* with *SLC2A1* in LIHC data from TCGA tumor. **E** The correlation of *SMAD5* with *SLC2A1* in liver data from GTEx. **F** Huh7 and HepG2 cells were treated with Ad-B4, Ad-GFP, Ad-siB4, or Ad-RFP to overexpression or silence BMP4, respectively. Western blotting was used to analyze the expression of SMAD5 and p-SMAD5. **G** ChIP assay was conducted to verify that potential SMAD5 bind sites to the SLC2A1 promoter in Huh7 and HepG2 cells

LDN193189 is a potent selective BMP type I receptor (BMP I) inhibitor that efficiently inhibits transcriptional activity of the BMP type I receptors ALK2 and ALK3. LDN193189 was used to treat Huh7 and HepG2 cells, PAS staining shown glycogen accumulation decreased after blocking canonical SMAD signaling pathway (Fig. 5A). TqPCR analysis showed the down expression of *GBE1*, *PYGM*, and *UGP2* (Fig. 5B). Interestingly, *GYS1* did not changed during LDN193189 treated, indicated that other important downstream signal of BMP4 such as p38 MAPK pathway may also participated in glycogen synthesis. We performed TqPCR analysis and demonstrated that BMP4 stimulation activated the canonical SMAD signaling pathway, as shown the increased expression of *SMAD*1/5/8 in the Huh7 and HepG2 cells (Fig. 5C). We analyzed the data from TCGA and GTEx and found a positive correlation of *SLC2A1* with *SMAD5* in LIHC (Fig. 5D) and liver (Fig. 5E) (http://guotosky.vip:13838/GPSA/. Accessed 13 May 2022). Western blotting analysis further demonstrated that BMP4 significantly increased the expression of SMAD5 and phosphorylated SMAD5 (p-SMAD5) (Fig. 5F). We performed the ChIP assay to assess these binding sites and found the specific binding of *SMAD5* to the promoter of *SLC2A1* in multiple positions (Fig. 5G). Taken together, these results indicate that BMP4 activates SMAD5 to facilitate the transcription of *SLC2A1* in Huh7 and HepG2 cells.

### BMP4 promotes glycogen accumulation in Huh7 cells by upregulating SLC2A1 through SMAD5 in vivo

**Fig. 6:**
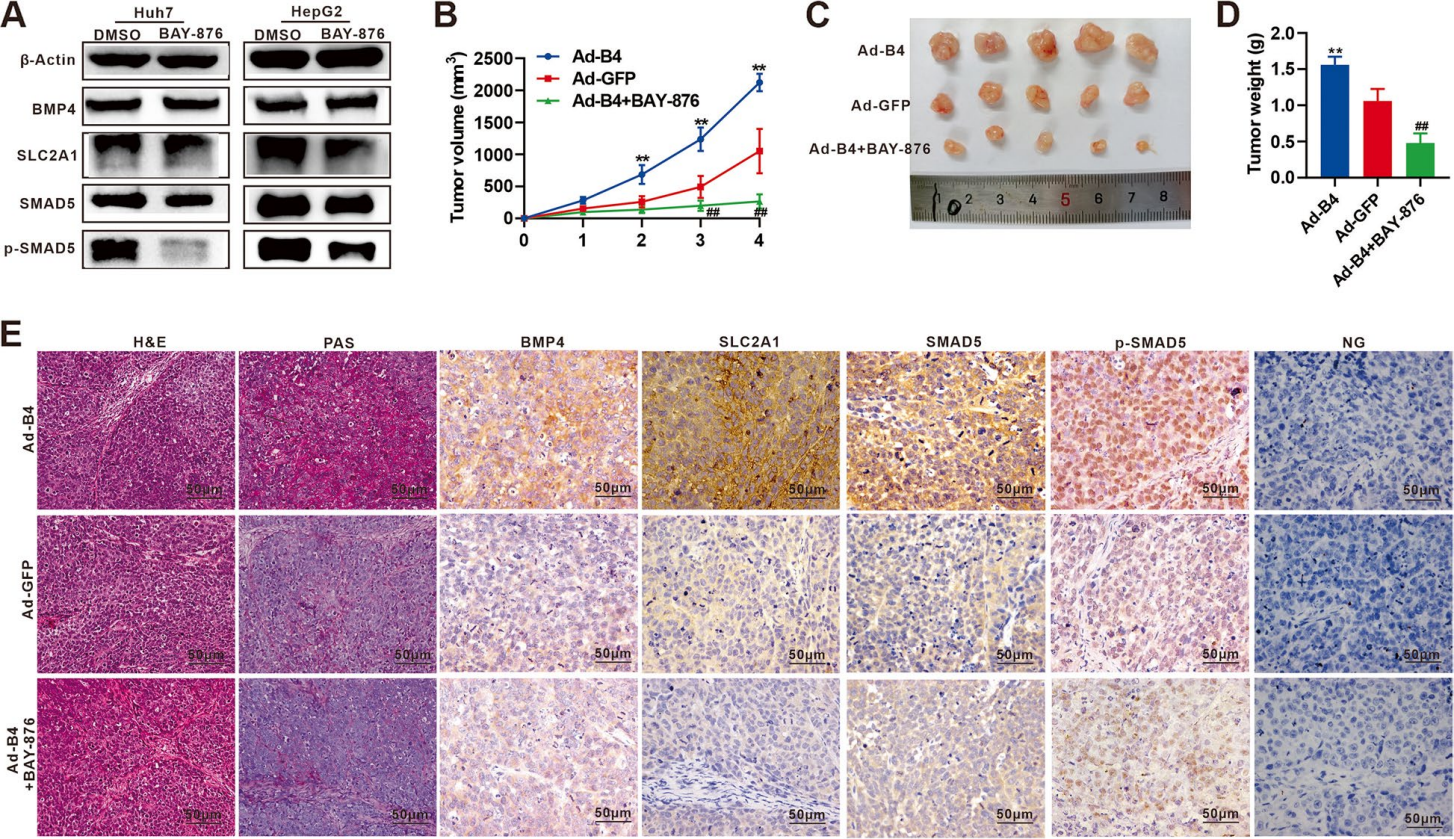
BMP4 reprograms hepatic glycogen accumulation and promotes tumor growth in vivo. **A** 1 μm BAY-876 or equal volume DMSO was used to treated Huh7 and HepG2 cells, and Western blot was used to analyze the expression of BMP4, SLC2A1, SMAD5, and p-SMAD5 in Huh7 and HepG2 cells after 72 h. **B** Huh7 cells were infected with Ad-B4 or Ad-GFP and subcutaneously injected into the flanks of athymic nude mice. The mice in Ad-B4 + BAY-876 group were administered through gavage with BAY-876, and the mice in Ad-B4 group and Ad-GFP group were administered through gavage with equal volume CMC. The average tumor volume was assessed from weeks 1 to 4 successively. **P < 0.01, ^##^*P* < 0.01, Ad-B4 group vs Ad-GFP group, Ad-B4 + BAY-876 group vs Ad-GFP group.** C** The size of subcutaneous tumor after 28 days of 6A. **D** The average tumor weight was assessed of 6A. **P < 0.01, ^##^*P* < 0.01, Ad-B4 group vs Ad-GFP group, Ad-B4 + BAY-876 group vs Ad-GFP group. **E** H & E, PAS, and IHC staining were used to check the morphology, glycogen accumulation, and the expression of BMP4, SLC2A1, SMAD5, and p-SMAD5 in Hu7 cells in vivo of **B**

We found that BAY-876 can significantly reduce the expression of SLC2A1 and p-SMAD5 in Huh7 and HepG2 cells in vitro (Fig. 6A). Using the subcutaneous xenograft tumor model, we found that BMP4 significantly promoted the growth of subcutaneous tumors, while BAY-876 significantly inhibited BMP4-induced subcutaneous tumor growth from weeks 2 to 4 (Fig. 6B). Detailed analysis indicates that BMP4 significantly promoted tumor volume and weight, which was significantly blunted by BAY-876 after 4 weeks (Fig. 6C & D). H & E staining analysis showed that BAY-876 inhibited the glycogen accumulation, while IHC staining assays revealed the high expression of SLC2A1, and p-SMAD5 was induced by BMP4 (Fig. 6E). Collectively, these findings demonstrate that BMP4 can promote glycogen accumulation in Huh7 cells by upregulating SLC2A1 through SMAD5 in vivo.

## Discussion

Glycogen synthesis takes place in the cytoplasm, where extracellular glucose is transported directly into the cell via a glucose transporter on the cell membrane [42]. The glycogen accumulation has been reported in many malignant tumors and cancer cells [43]. In this study, we found that glycogen accumulation occurs in hepatocellular carcinoma tissues, compared with that in the adjacent normal tissue, suggesting that abnormal glycogen accumulation may play an important role in promoting hepatocellular carcinoma.

BMPs are members of the transforming growth factor β (TGF-β) family. To date, about 20 BMP family members have been identified. The receptors on the surface of the cell membrane of BMPs are mainly classified into BMPRI type receptors and BMP type II receptors, among which the type I receptors include ALK1, ALK2, ALK3, and ALK6, while the type II receptors mainly include ActRIIa, ActRIIb, and BMPRII. Mature BMP proteins are disulfide-linked homo- or heterodimers that are secreted and bound to receptors on the surface of target cells. BMP4 initiates downstream signal by binding to type I or type II serine/threonine kinase receptors and forming a heterotetrameric complex. In the classical pathway, activated BMPR I receptors phosphorylate R-SMADs (SMAD1/5/8), which bind to Co-SMADS (SMAD4) to form a complex that acts as a transcription factor in the nucleus and can bind to the promoter of target genes and participate in the regulation of target gene transcription [34, 44]. In this study, we found BMP4 facilitates the transcription of SLC2A1 via the canonical SMAD signaling pathway. SLC2A1 (GLUT1) is a member of the glucose transporter family and is a key regulatory element that mediates the transport of extracellular glucose across the membrane into the cell [45]. Meanwhile, cancer cells express SLC2A1 at much higher levels on their cell membrane than normal cells, thereby selectively and excessively take up more glucose and provide a basis for promoting glycogen synthesis and accumulation [46]. SLC2A1 is the most extensively studied in a wide range of malignancies, like such as prostate, ovarian cancer, and breast as well as bladder cancers, while abnormally high GLUT1 expression correspondence with the malignant phenotype of tumors [47–50]. Our work further confirmed the similar function and new mechanism of SL2A1 in glycogenesis of liver cancer.

BMP signaling plays an essential function during carcinogenesis and progression of various human malignancies [51]. It was reported that BMP4 was highly expressed in hepatocellular carcinoma and promoted the malignant phenotype of the tumor [30, 51–54]. Upregulation of BMP4 and BMPR1A in HCC was shown to promote proliferation and metastasis of hepatocellular carcinoma cells through activation of the MEK/ERK signaling pathway [55]. Liver cancer-associated fibroblasts (CAFs) were shown to secrete cytokines under the activation of endogenous and exogenous BMP4 to enhance invasiveness of liver cancer cells [54]. BMP4-induced epithelial mesenchymal transition through upregulation of ID2 and promoted metastasis in hepatocellular carcinoma, which was associated with poor prognosis in patients with 420 paired HCC study [34]. Furthermore, BMP4-mediated autophagy activation may contribute to HCC proliferation through the JNK1/Bcl2 signaling pathway [56]. In this study, we found BMP type I receptor (BMP I) inhibitor LDN193189 and SLC2A1 inhibitor BAY-876 can effectively inhibit glycogenesis and glycogenolysis genes in HCC cells, suggesting that BMP4 plays a regulatory role through SMAD signaling pathway. Interestingly, *GYS1* did not changed during LDN193189 treated, indicated that other important downstream signal of BMP4 such as p38 MAPK pathway may also participated in glycogen synthesis [57].

Our study reveals that BMP4 is highly expressed in liver cancer tissues and promotes glycogen synthesis via the canonical SMAD signaling pathways. TqPCR analysis also show that the glycogenesis-related genes *GBE1*, *GYS1*, *UGP2*, and glycogenolysis-related gene *PYGM* are significantly upregulated in response to BMP4 stimulation, while downregulation of BMP4 reverses the outcomes. Through upregulation of gluconeogenesis and glycogenolysis-related enzymes via BMP4 in hepatocellular carcinoma cells, we found that metabolic adaptation of cells is a dynamic process, HCC cells required the generation of intracellular glycogen for storing energy and needed to consumption of intracellular glycogen for survival. BMP4 shows a crucial role in glucose metabolism, and its important role in energy metabolism in liver cancer deserves further exploration.

## Conclusion

As shown in Fig. S3, in this study, we blocked the function of SLC2A1 using BAY-876 and discovered that it could suppress the expression of gluconeogenesis and glycogenolysis-related genes in hepatocellular carcinoma cells. Meanwhile, we found overexpression of Ad-B4 could not rescue the metabolic alterations caused by BAY-876, suggesting that SLC2A1 functions downstream of BMP4 through transcription upregulation of SLC2A1 expression through SMAD5 signaling.

## Supplementary Information

**Additional file 1: Table S1.** BMP4 and SLC2A1 IHC score results. **Table S2.** List of TqPCR primers. **Table S3.** List of PCR primers for ChIP assay.

**Additional file 2: Fig. S1.** The original images of WB in Figs. 2, 3, 5 and 6. **Fig. S2.** **A** The expression of BMP4 and SLC2A1 in Lo2, Huh7, MHCC 97H, HepG2 and Hep3B were assessed by Western blotting. **B** Huh7 and HepG2 were infected with Ad-B4, Ad-GFP, Ad-siB4 or Ad-RFP, respectively, and fluorescence images were taken at 36h. **C** Huh7 and HepG2 were infected with Ad-B4, Ad-GFP, Ad-siB4 or Ad-RFP respectively, and TqPCR analysis was used to evaluate the expression of *SLC2A1*, *SLC2A2*, *SLC2A3 *and* SLC2A4* at 24h and 48h. “**” *P *< 0.01, “*” *P *< 0.05, Ad-B4 group vs Ad-GFP group, Ad-siB4 group vs Ad-RFP group. **D** Huh7 and HepG2 were treated with 1μm BAY-876 or equal volume DMSO respectively, and TqPCR analysis was used to evaluate the expression of *SLC2A1*, *SLC2A2*,* SLC2A3 *and* SLC2A4* at 36h. “**” *P *< 0.01, “*” *P *< 0.05, BAY-876 group vs DMSO group. **Fig. S3.** The graphic abstract of this research.

## Data Availability

Not applicable.

## Abbreviations

BMP4: Bone morphogenetic protein 4
Ad-B4: Adenoviral vector expressing human BMP4
Ad-GFP: Adenoviral vector expressing the green fluorescent protein (GFP)
Ad-siB4: Adenoviral vector expressing three siRNAs that silence human Bmp4
Ad-RFP: Adenoviral vector expressing the red fluorescent protein (RFP)
H & E: Hematoxylin and eosin
IHC: Immunohistochemistry
IF: Immunofluorescence
TqPCR: Touchdown quantitative real-time PCR
ChIP: Chromatin immunoprecipitation
SLC2A1: Solute carrier family 2 member 1
GBE1: 1,4-Alpha-glucan branching enzyme 1
GYS1: Glycogen synthase 1
UGP2: UDP-glucose pyrophosphorylase 2
PYGM: Glycogen phosphorylase, muscle associated
SMAD1/5/8: SMAD family member 1/5/8
