# Supplementary material for: BMP4 upregulates glycogen synthesis through the SMAD/SLC2A1 (GLUT1) signaling axis in hepatocellular carcinoma (HCC) cells
Source: Cancer Metab. 2023 Jul 13;11:9. doi: 10.1186/s40170-023-00310-6 (PMC10339511; doi:10.1186/s40170-023-00310-6)
Supplement: Supplementary file 1 — Additional file 1: Table S1. BMP4 and SLC2A1 IHC score results. Table S2. List of TqPCR primers. Table S3. List of PCR primers for ChIP assay. [file 40170_2023_310_MOESM1_ESM.zip › Table S1.docx]

| BMP4 IHC score  Specimen number | HCC | Paracancer | SLC2A1 IHC score  Specimen number | HCC | Paracancer |
| --- | --- | --- | --- | --- | --- |
| No.1 | 2 | 4 | No.1 | 2 | 4 |
| No.2 | 4 | 2 | No.2 | 5 | 2 |
| No.3 | 4 | 4 | No.3 | 5 | 4 |
| No.4 | 3 | 1 | No.4 | 4 | 2 |
| No.5 | 4 | 1 | No.5 | 5 | 1 |
| No.6 | 3 | 4 | No.6 | 3 | 3 |
| No.7 | 1 | 1 | No.7 | 3 | 2 |
| No.8 | 1 | 1 | No.8 | 2 | 2 |
| No.9 | 3 | 2 | No.9 | 4 | 3 |
| No.10 | 3 | 3 | No.10 | 4 | 3 |
| No.11 | 4 | 3 | No.11 | 3 | 3 |
| No.12 | 5 | 4 | No.12 | 4 | 4 |
| No.13 | 5 | 2 | No.13 | 4 | 2 |
| No.14 | 1 | 1 | No.14 | 2 | 1 |
| No.15 | 4 | 4 | No.15 | 4 | 3 |
| No.16 | 3 | 1 | No.16 | 3 | 2 |
| No.17 | 1 | 4 | No.17 | 2 | 4 |
| No.18 | 4 | 1 | No.18 | 4 | 1 |
| No.19 | 3 | 3 | No.19 | 3 | 3 |
| No.20 | 4 | 3 | No.20 | 4 | 2 |
| No.21 | 3 | 1 | No.21 | 2 | 1 |
| No.22 | 1 | 4 | No.22 | 1 | 4 |
| No.23 | 4 | 1 | No.23 | 3 | 1 |
| No.24 | 3 | 2 | No.24 | 2 | 2 |
| No.25 | 4 | 4 | No.25 | 4 | 4 |
| No.26 | 4 | 4 | No.26 | 4 | 2 |
| No.27 | 4 | 4 | No.27 | 4 | 3 |
| No.28 | 3 | 4 | No.28 | 3 | 2 |
| No.29 | 3 | 3 | No.29 | 3 | 4 |
| No.30 | 3 | 2 | No.30 | 3 | 2 |
| No.31 | 3 | 2 | No.31 | 3 | 1 |
| No.32 | 2 | 1 | No.32 | 2 | 2 |
| No.33 | 3 | 2 | No.33 | 3 | 3 |
| No.34 | 3 | 1 | No.34 | 3 | 2 |
| No.35 | 1 | 2 | No.35 | 2 | 2 |
| No.36 | 3 | 3 | No.36 | 3 | 3 |
| No.37 | 4 | 3 | No.37 | 4 | 3 |
| No.38 | 1 | 4 | No.38 | 2 | 4 |
| No.39 | 4 | 5 | No.39 | 4 | 3 |
| No.40 | 4 | 1 | No.40 | 4 | 1 |
