# Supplementary figures and images for: BMP4 upregulates glycogen synthesis through the SMAD/SLC2A1 (GLUT1) signaling axis in hepatocellular carcinoma (HCC) cells
Source: Cancer Metab. 2023 Jul 13;11:9. doi: 10.1186/s40170-023-00310-6 (PMC10339511; doi:10.1186/s40170-023-00310-6)

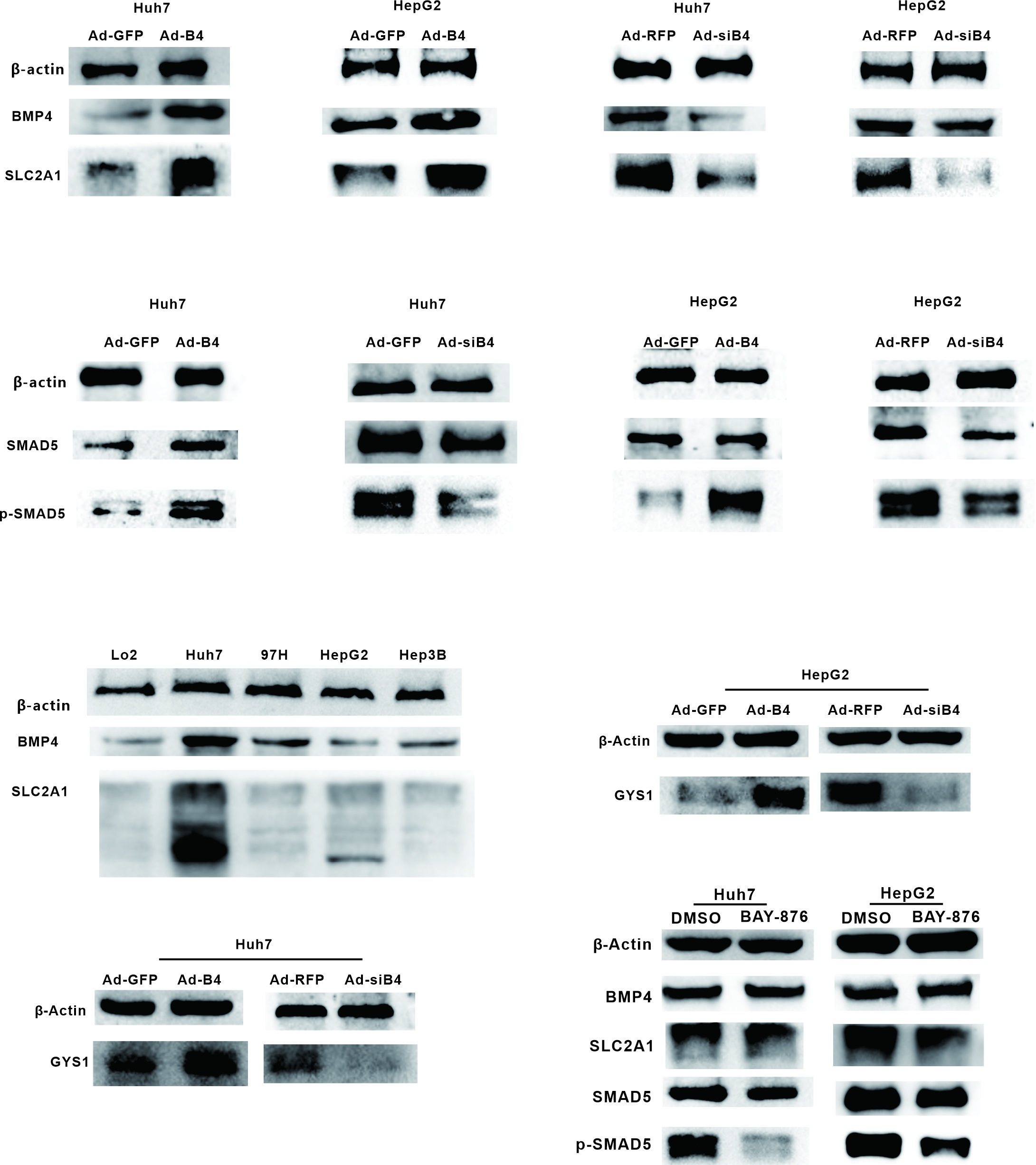

Supplement: Supplementary file 2 — Additional file 2: Fig. S1. The original images of WB in Figs. 2, 3, 5 and 6. Fig. S2. A The expression of BMP4 and SLC2A1 in Lo2, Huh7, MHCC 97H, HepG2 and Hep3B were assessed by Western blotting. B Huh7 and HepG2 were infected with Ad-B4, Ad-GFP, Ad-siB4 or Ad-RFP, respectively, and fluorescence images were taken at 36h. C Huh7 and HepG2 were infected with Ad-B4, Ad-GFP, Ad-siB4 or Ad-RFP respectively, and TqPCR analysis was used to evaluate the expression of SLC2A1, SLC2A2, SLC2A3 and SLC2A4 at 24h and 48h. “**” P < 0.01, “*” P < 0.05, Ad-B4 group vs Ad-GFP group, Ad-siB4 group vs Ad-RFP group. D Huh7 and HepG2 were treated with 1μm BAY-876 or equal volume DMSO respectively, and TqPCR analysis was used to evaluate the expression of SLC2A1, SLC2A2, SLC2A3 and SLC2A4 at 36h. “**” P < 0.01, “*” P < 0.05, BAY-876 group vs DMSO group. Fig. S3. The graphic abstract of this research. [file 40170_2023_310_MOESM2_ESM.zip › Figure S1.jpg]

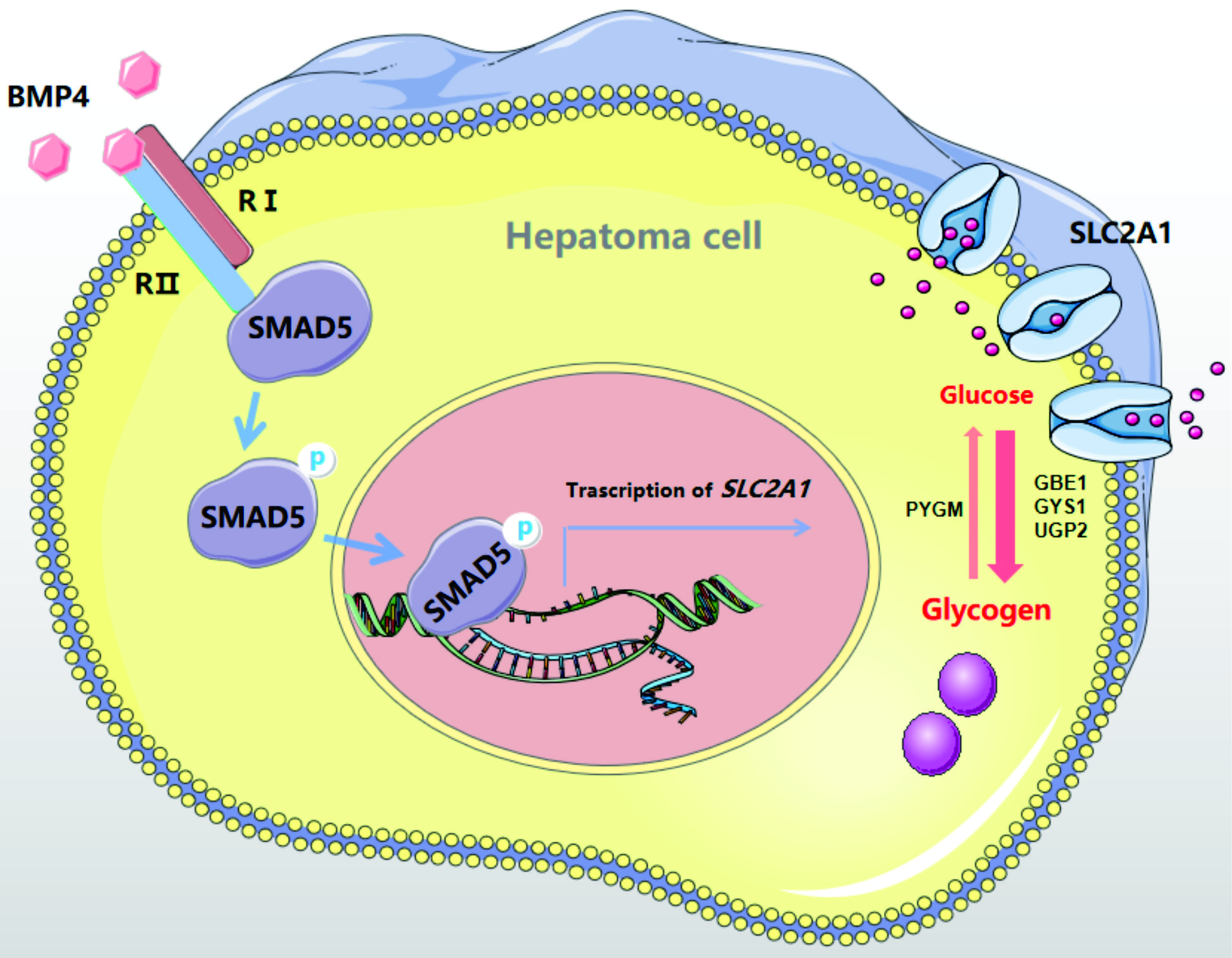

Supplement: Supplementary file 2 — Additional file 2: Fig. S1. The original images of WB in Figs. 2, 3, 5 and 6. Fig. S2. A The expression of BMP4 and SLC2A1 in Lo2, Huh7, MHCC 97H, HepG2 and Hep3B were assessed by Western blotting. B Huh7 and HepG2 were infected with Ad-B4, Ad-GFP, Ad-siB4 or Ad-RFP, respectively, and fluorescence images were taken at 36h. C Huh7 and HepG2 were infected with Ad-B4, Ad-GFP, Ad-siB4 or Ad-RFP respectively, and TqPCR analysis was used to evaluate the expression of SLC2A1, SLC2A2, SLC2A3 and SLC2A4 at 24h and 48h. “**” P < 0.01, “*” P < 0.05, Ad-B4 group vs Ad-GFP group, Ad-siB4 group vs Ad-RFP group. D Huh7 and HepG2 were treated with 1μm BAY-876 or equal volume DMSO respectively, and TqPCR analysis was used to evaluate the expression of SLC2A1, SLC2A2, SLC2A3 and SLC2A4 at 36h. “**” P < 0.01, “*” P < 0.05, BAY-876 group vs DMSO group. Fig. S3. The graphic abstract of this research. [file 40170_2023_310_MOESM2_ESM.zip › figure S3.jpg]

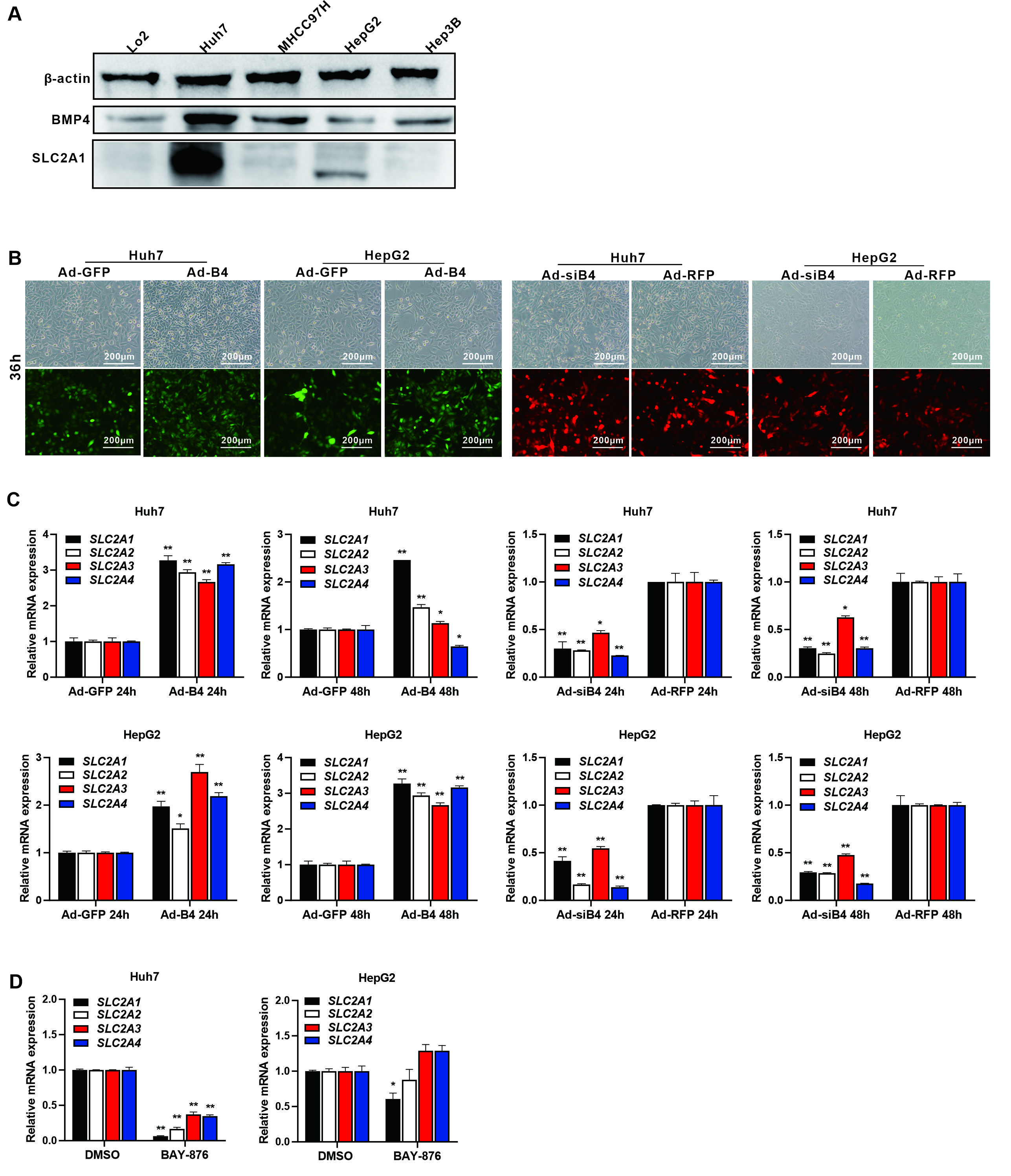

Supplement: Supplementary file 2 — Additional file 2: Fig. S1. The original images of WB in Figs. 2, 3, 5 and 6. Fig. S2. A The expression of BMP4 and SLC2A1 in Lo2, Huh7, MHCC 97H, HepG2 and Hep3B were assessed by Western blotting. B Huh7 and HepG2 were infected with Ad-B4, Ad-GFP, Ad-siB4 or Ad-RFP, respectively, and fluorescence images were taken at 36h. C Huh7 and HepG2 were infected with Ad-B4, Ad-GFP, Ad-siB4 or Ad-RFP respectively, and TqPCR analysis was used to evaluate the expression of SLC2A1, SLC2A2, SLC2A3 and SLC2A4 at 24h and 48h. “**” P < 0.01, “*” P < 0.05, Ad-B4 group vs Ad-GFP group, Ad-siB4 group vs Ad-RFP group. D Huh7 and HepG2 were treated with 1μm BAY-876 or equal volume DMSO respectively, and TqPCR analysis was used to evaluate the expression of SLC2A1, SLC2A2, SLC2A3 and SLC2A4 at 36h. “**” P < 0.01, “*” P < 0.05, BAY-876 group vs DMSO group. Fig. S3. The graphic abstract of this research. [file 40170_2023_310_MOESM2_ESM.zip › figure S2.jpg]
